# Development, safety and efficacy of a novel circular-irrigated deca-channel mapping and ablation catheter for pulmonary vein isolation

**DOI:** 10.1186/s12872-018-0886-1

**Published:** 2018-08-15

**Authors:** Xiang-Fei Feng, Mu Chen, Jian Sun, Jie Sun, Bo Liang, Yi-Yong Sun, Yi-Gang Li

**Affiliations:** 1 Department of Cardiology, Xinhua Hospital, School of Medicine, Shanghai Jiao Tong University, 1665#, KongJiang Road, Shanghai, 200092 China; 2 Shanghai MicroPort EP MedTech Co., Ltd, Building #28, Lane 588, Tianxiong Road, Shanghai, China

**Keywords:** Atrial fibrillation, Catheter ablation, Circular-irrigated deca-channel ablation catheter, Pulmonary vein isolation, Design requirement

## Abstract

**Background:**

Pulmonary vein isolation (PVI), a cornerstone for catheter ablation of atrial fibrillation (AF), remains a complex and time-consuming procedure. Present study introduces a novel, circular-irrigated, deca-channel mapping and ablation catheter (CIDMA), describes the in vitro test results on feasibility, safety, and acute efficacy of the CIDMA catheter.

**Methods:**

An assembled CIDMA catheter was subjected to a number of in vitro tests. With this catheter, ablation procedures were first performed in a pig’s myocardial strips in vitro to determine the effects in unipolar or bipolar configuration.

**Results:**

Three catheters were assembled. The adjustable circular diameter was changed from initial state of 32.41 ± 0.61 mm into controlled state of 28.61 ± 0.47 mm (*P* = 0.013). In the plastic model, the push-ability, torque-ability, and kink resistance of CIDMA catheter were shown to be satisfactory.

In vitro, our findings showed that ablation could produce obvious ablation lesions, and unipolar ablation (at length, width and depth of 5.0 ± 1.3, 4.6 ± 0.7, and 4.2 ± 0.6 mm, respectively) was more effective than bipolar (at length, width and depth of 2.8 ± 0.2, 4.2 ± 0.5, and 2.3 ± 0.4 mm, respectively) (*P* < 0.01).

**Conclusions:**

In vitro, our preliminary data suggest that the CIDMA catheter produced optimal ablation lesions, especially in the unipolar ablation mode. Future in vivo animal and clinical studies are warranted to test the efficacy of this catheter in real-world scenario.

## Background

Atrial fibrillation (AF) is currently the most common arrhythmia encountered in clinical practice, and pulmonary vein isolation (PVI) has been established as a standard procedure for the treatment of symptomatic and drug refractory AF, and a single-tip ablation catheter is usually used for creating linear lesions around ipsilateral pulmonary veins (PVs) during PVI [1, 2]. Despite the routine use of three-dimensional (3D) mapping systems, manual point-by-point ablation can still be a complex and time consuming procedure, and ablation lesions can vary considerably depending on the applied catheter contact force, orientation, size, and energy parameters [3]. Moreover, creation of a durable, contiguous transmural ablation line around the PVs is challenging with single-tip catheters, and the long-term efficacy remains far away from satisfactory [4].

Therefore, various specialized devices, utilizing different energy forms and catheter designs, have been developed to simplify this procedure [5–7]. The pulmonary vein ablation catheter (PVAC) is the multielectrode mapping and ablation catheter used for PVI on a routine basis. But up to now, PVAC was shown to have several disadvantages such as using nonirrigated energy delivery, and the lack a so-called single-shot capacity, and a 3D mapping capacity [8, 9].

The nMARQ catheter (Biosense Webster, Inc., Diamond Bar, Ca, USA), is an effective single-shot device [10]. However, the device also presented with some safety concerns arising from the severe device-related complications [11, 12], and ultimately leading to the interim recall. So, the single-tip, point-by-point radiofrequency (RF) ablation system remains as the most widespread technology applied in the field of AF ablation [13].

To perform the ‘en bloc’ ablation of the ipsilateral PVs, and reduce the procedure time, a novel, circular-irrigated, deca-channel mapping and ablation catheter (CIDMA) is designed and developed by Micro Port EP MedTech Co. Ltd. (Shanghai, China). This catheter is developed and could be used as a multi-selective catheter for MRI-guided cardiovascular interventions [14]. Comparing with nMARQ catheter, this catheter could conveniently adapt to PV anatomy during the PVI procedure due to improved technical and morphological designs. Moreover, the added maneuver ability of the distal tip from this catheter may improve the efficiency and safety of the whole PVI procedure.

The present paper will first introduce the main development requirements and initial laboratory test results of CIDMA catheters, and then describe the initial in vitro test results of CIDMA catheter.

## Methods

The requirements of the CIDMA catheter were initiated and designs of the CIDMA were approved and updated, then the CIDMA catheters were assembled, and the characteristics were verified. Finally, we evaluated the performance of the newly developed CIDMA catheter in vitro to evaluate the feasibility, safety, and acute efficacy of the CIDMA catheter.

**Fig. 1:**
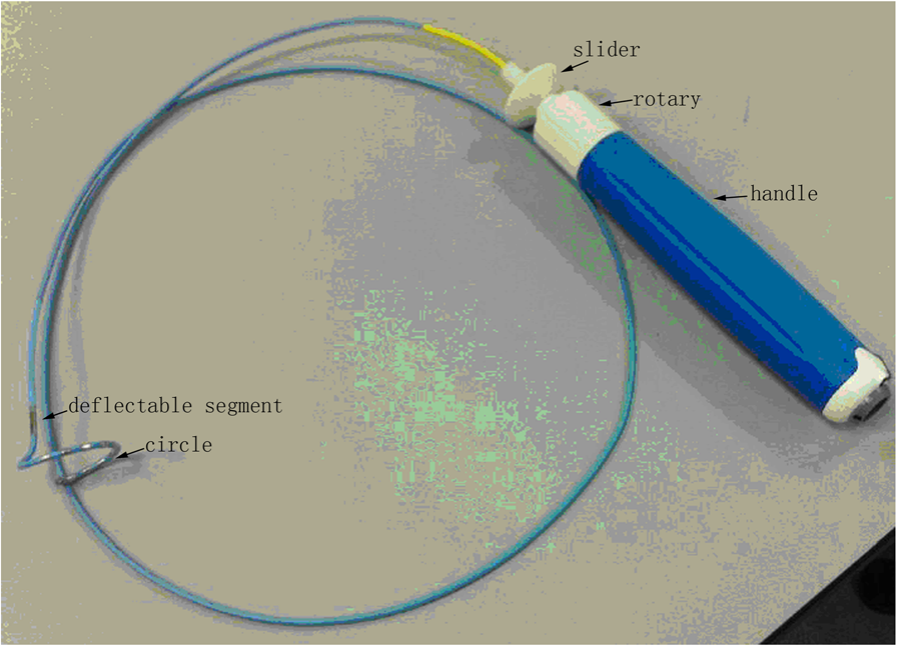
The picture of a CIDMA catheter

This study referenced to previously published works [9, 14–16]. A CIDMA catheter includes connected in sequence, a distal portion, a main body portion and a control handle (Fig. 1). The distal portion can, at least partially, reversibly change into a circumferential configuration form with a lower contour configuration, the upper portion of which is equipped with a plurality of electrodes used for ablation.

### Animal experiment

We adhered to ARRIVE guidelines for pre-clinical animal studies. Animal care and handling procedures were approved by the Institutional Animal Care and Use Committee of XinHua Hospital (approval number: XHEC-F-2017-023) in accordance with the Guide for the Care and Use of Laboratory Animals published by the National Institutes of Health (NIH Publication No.85–23, revised 1996).

### Design requirements

#### Mapping and ablation requirements

Using an irrigated multi-electrode electro-anatomically guided mapping and ablation catheter might have more advantages over the currently available ablation systems. Therefore, the first requirement is to develop a novel ablation system, which has both decapolar mapping and ablation capacities, and possesses both the irrigation and electro-anatomic mapping features.

To meet this requirement, the distal plurality portions of the CIDMA catheter should be designed as the helical in form, thus being able to ablate targets with plurality features to form a desired shape of ablation. In addition, the inner cavity of the catheter should have a coolant liquid perfusion flow path and could be used to quickly reduce the temperature of the electrode according to command, and each electrode can be turned on or off at any time of RF delivery individually.

#### Deflectable requirement

The catheter should have a deflectable segment and an adjustable circle, which can then encircle the whole ipsilateral superior or inferior PVs, compatible to 3D mapping system (Ensite Velocity system, St Jude Medical, Inc).

#### Handle maneuverability

The handle portion of the catheter should be able to drive deflectable segment and variable circle independently from the tool’s proximal extremity to meet the navigation tasks, the tip should be driven independently without affecting the orientation function of the handle portion.

#### Compatibility

The catheter should be compatible with conventional instruments, such as guide sheaths and 3D mapping system. Peripheral interventions mostly require the use of 0.032 in-guide wires (0.89 mm diameter) together with long, 8.5Fr (2.83 mm) diameter Swarts sheaths. In order to meet this compatibility, the diameter of the catheter should be based on the size of these commercial instruments. In addition to the dimension requirements and 3D inducting requirement, the catheter should also possess other important features such as suitable stiffness, pushability, and torque control.

### Design

#### Irrigated electrodes design of the CIDMA catheter

Perfusion fluid channels are incorporated in the main body portion, and the control handle. The distal portion includes a perfusion fluid lumen, a lateral wall equipped with an opening. Furthermore, a luer is implemented at the tail end of the handle, which serves as a path between the catheter lumen and outside.

**Fig. 2:**
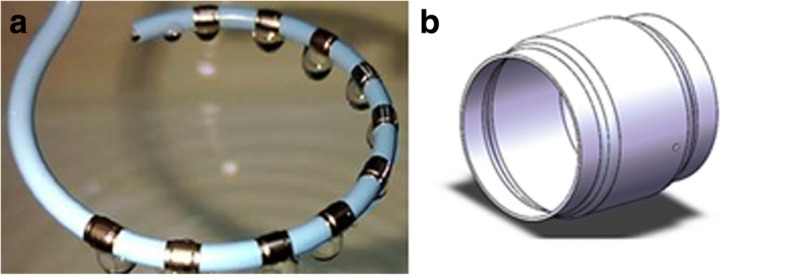
The picture of variable circle and irrigated electrode. **a** top-view of a variable circle; **b** side-view of a irrigated electrode

On a variable circle of the distal portion, 10 separate, openly irrigated electrodes (Platinum electrode length 3.0 mm, spacing 4 mm, maximum diameter 8.4 French) are arranged (Fig. 2a). Intracardiac signals could be obtained by five bipolar recordings through adjacent electrode pairs. Each of the electrodes possesses a thermocouple and 10 perfusion apertures (0.1 mm) arranged in two rows at both ends of the electrode for irrigation (Fig. 2b).

Each electrode surrounds at least partially the circumferentially affixed arrangement of the distal portion and forms a perfusion fluid chamber between itself and perfusion fluid lumen. Each perfusion aperture is fluidly connected to a perfusion fluid source by means of a chamber, a corresponding opening, a perfusion fluid lumen, and a perfusion fluid channel.

#### Design of the main body portion

As described in detail previously [14], the main body portion is the shaft of the catheter (115 cm long, 2.52 mm outside diameter), which is made of polyether ether ketone (PEEK) tube, a biocompatible material with the stiffest polymer and high tensile strength, torqueability, and pushability. The deflectable segment or the adjustable circle could be actuated by a 0.12 mm diameter pull-wire that is attached at the most distal part of its corresponding segment. A nitinol rod (10 cm long, 0.52 mm diameter) is placed in an additional groove, milled along proximal segment of the PEEK tube, to provide additional stiffness. Polyimide is placed around the PEEK tube to keep the wires and the nitinol rod in the grooves.

#### Distal end portion design of the CIDMA catheter

To mimic the shapes of the Lasso catheter, one deflectable segment and one variable circle are constituted at the distal end of the catheter. The steering mechanisms are placed at the handle (Fig. 1); the catheter can thus be deflected unidirectionally, while the diameter of the loop can be varied from 20 to 35 mm. The maximum relative angle between the two links is 180° to ensure that water could be flush through the catheter lumen without obstruction. The links or hinges were manufactured by laser cutting technology [14].

#### Design of the handle portion

The handle portion is composed of three segments: distal segment, middle segment, and proximal segment. The distal segment of the handle portion is like a slider, which could drive the end of the wire of the deflectable segment. Therefore, the handle could precisely control the movement of the deflectable distal segment by applying suitable tension on the pull-wires. Translating the slider backward actuates the segment in one direction while pushing the slider front actuates the same segment in the opposite direction.

The middle segment is a rotary, rotating this segment could alter the diameter of the variable circle. A holding mechanism ensures that the shape of the catheter would stay fixed when the slider or rotary is released.

#### Energy supply of the CIDMA catheter

As described in detail previously [9], the generator is a multichannel RF generator capable of delivering independently unipolar or bipolar RF energy to a maximum of 10 electrodes simultaneously. In unipolar ablation configuration, current flow is provided between the selected electrodes and an indifferent electrode attached to the back, while during bipolar ablation, current flows between adjacent electrodes.

A temperature controlled manner is applied, and the maximum energy level can be controlled from 1 to a maximum of 25 W in unipolar and up to 15 W in bipolar ablation mode. All parameters such as temperature, impedance and power levels for every single electrode are displayed on a separate screen. To irrigate all electrodes, a cooling pump is used to provide a continuous flow of heparinized saline fluid with the maximum speed of 60 ml/min.

### Statistics

Descriptive statistics were used to report patient characteristics. Continuous variables with normal distribution were reported as mean ± SD. Median (25th to 75th percentiles) was used with abnormal distribution. Percentages were used to report categorical variables. All statistical analyses were performed in SPSS software (version 22.0, SPSS Inc., Chicago, Illinois).

## Results

### Catheter verification

According to the aforementioned design, three CIDMA catheters were assembled (Fig. 1) and the deforming forces of catheter should not exceed the corresponding elastic limit of the materials used to manufacture catheter [17]. Each assembled catheter was subjected to a number of test described below and should meet design requirements.

#### Bending load of the catheter shaft

Bending load could be used to assess relative catheter stiffness, one important aspect of catheter design [18]. The catheter shaft was placed on the positioning dip in water and the size of the bending load was measured by the movement of the stretching machine. Test results were shown in Table 1. From Table 1, we could see that as the soaking time lengthened, the bending load gradually became smaller, from initial 0 min 2.9331 ± 0.1774 Newton, to 30 min 1.8654 ± 0.1355 Newton, and to 240 min 1.5469 ± 0.1767 Newton (*P* = 0.018).

**Table 1:** The characteristics of the CIDMA catheter

Sample	The bending load of shaft soaking in water (N)			The diameter of adjustable circular (mm)		The diameter in loop (mm)	The bending force of handle (N)
	In water 0 min	In water 30 min	In water 240 min	Initial state	Controlled state		
1#	3.1379	2.008	1.7442	31.73	28.77	2.35	13.53
2#	2.8277	1.7384	1.4932	32.92	28.98	2.33	13.75
3#	2.8336	1.8497	1.4032	32.57	28.09	2.25	13.55
Mean ± SD	2.9331 ± 0.1774	1.8654 ± 0.1355	1.5469 ± 0.1767	32.41 ± 0.61	28.61 ± 0.47	2.31 ± 0.05	13.61 ± 012
*P* value	0.018			0.013		–	–

#### The adjustable circle diameter of the CIDMA catheter

We measured the adjustable circle diameter of the CIDMA catheter with a special gage in the original state and control state respectively. Test results were shown in Table 1 and the diameter of adjustable circular could be changed significantly, from initial state 32.41 ± 0.61 mm into controlled state 28.61 ± 0.47 mm (*P* = 0.013).

#### The outside diameter measurement of the CIDMA catheter

We measured the catheter diameter in loop segment with a special gage. The test results were shown in Table 1. The diameter in loop segment was 2.31 ± 0.05 mm, which comply with the design requirements.

#### The bending force of the handle

The handle of the CIDMA catheter is equipped with position control device; the handle bending force could be measured by the force measurement device. Test results were shown in the Table 1. The bending force of the handle was 13.61 ± 012 Newton, which comply with the design requirements.

#### Push performance of the CIDMA catheter

The CIDMA catheter was placed in the plastic transparent vascular and heart model. In the model setting, the CIDMA catheter successfully passed the inferior vena cava and reached the right atrium, right ventricle, common pulmonary artery trunk through the right femoral vein access. By minimizing the diameter, bending tip, pushing and rotating handle, the CIDMA catheter could enter left atrium through Swartz sheaths smoothly, and enter into pulmonary vein subsequently.

### Animal experimental evaluation of the CIDMA catheter

Four healthy farm dogs weighing 20–35 kg, and two healthy farm pigs weighing 40–60 kg were included in the study. As described previously [15], all animals were fed healthy normocholesterolemic food once daily. At the end of the study, animals were euthanized under isoflurane anesthesia with an intravenous overdose of potassium chloride and the entire carcass was submitted for pathological examinations by designated persons.

#### Electroanatomic mapping capability

In order to evaluate electroanatomic mapping capability of the catheter, the CIDMA catheter was tested in a general anesthetized dog under real-time 3D anatomic mapping with Ensite Velocity system (St Jude Medical, Inc., USA). As described previously [14], a 12 Fr introducer was placed in the right femoral vein and the procedure was performed under fluoroscopic and angiographic guidance. A medical doctor, experienced with the manipulation of endovascular instrument and of 3D anatomic mapping, performed the manipulations.

The circled structure was visible under real-time 3D anatomic mapping, and had a high 3D inducting accuracy (0.6~ 1.5 mm), which was minimally interfered by the external circumference. During the mapping tasks, it was possible to manipulate the circled structure in the inferior vena cava and right atrium.

#### Ablation results of the CIDMA catheter

In order to evaluate the ablation efficacy of the catheter, ablation procedures were first performed in a pig’s myocardial strips in vitro. In vitro, RF energy was applied in a bipolar configuration mode through adjacent electrode pairs and then in unipolar configuration mode between the electrodes and an indifferent electrode.

Fresh hearts were isolated from healthy pigs using methods described previously [16]. The left and right ventricles were removed and sliced into approximately 4 * 4 *2 cm slices incorporating the endocardium. Only slices with relatively smooth endocardial surfaces were utilized. Pig myocardial strips were inserted into a specially designed polyurethane chamber. The CIDMA catheter was inserted through a 25-cm glass rod positioned perpendicular to the myocardial tissue. This tubing provided a smooth uniform track to guide the catheter and insured the consistent perpendicular contact with the endocardial surface of the tissue.

Under a temperature controlled manner, energy level can be regulated automatically. Maximum of parameters were displayed as follows in unipolar or bipolar ablation mode (Table 2). Each electrode can be turned on or off at any time of RF delivery individually.

**Table 2:** The ablation parameters set of animal experiment in vivo

	Contact force	Perfusion flow	Power	Time	Temperature
Unipolar ablation	——	60 ml/min	20 W	45S	45 °C
Bipolar ablation	——	60 ml/min	15 W	45S	45 °C

##### Unipolar ablation in vitro

**Fig. 3:**
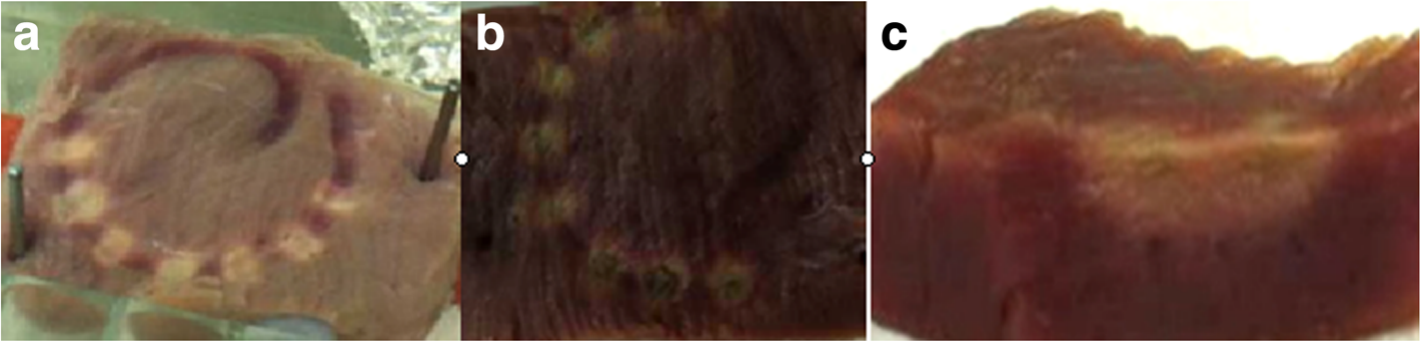
The photographs of ablation on tissue (pig’s myocardial strip) in vitro. Uniformity in the shape of ablation lesions was seen around the electrodes. **a** top-view of ablated tissue post unipolar ablation; **b** top-view of ablated tissue post bipolar ablation; **c** bipolar ablation lesion depth on ablated tissue bisection plane

After ablation, the results showed that unipolar ablation could uniformly produce obvious ablation lesions with a length, width and depth of 5.0 ± 1.3, 4.6 ± 0.7, and 4.2 ± 0.6 mm, respectively (Table 3, Fig. 3a). On ablated tissue bisection plane, there were two distinct regions of ablated tissue surrounded by non-ablated tissue and separated by an approximately 2-mm non-ablated gap, and the gross pathology ablation lesion depth was between 3 mm and 5 mm (Fig. 3).

**Table 3:** The ablation characteristics of animal experiment in vivo (*n* = 4)

	values	The number of related electrodes (uni/bipolar,10/10)	unipolar ablation (*n* = 2)	bipolar ablation (*n* = 2)	*P* value
Ablation characteristics	Last power (W)	10/10	19.6 ± 1.3	15.0 ± 0.0	0.000
	max power (W)	10/10	20.0 ± 0.0	15.0 ± 0.0	0.000
	Full power time (S)	10/10	11.7 ± 2.1	7.1 ± 2.5	0.000
	Full temperature time (S)	1/0	30.0 ± 0.0	–	–
	initial temperature (°C)	10/10	37.4 ± 0.5	36.8 ± 0.4	0.003
	final temperature (°C)	10/10	40.9 ± 2.0	37.1 ± 2.8	0.000
	initial resistance (Ω)	10/10	175.0 ± 8.2	174.3 ± 22.5	0.926
	final resistance (Ω)	10/10	140.8 ± 9.5	164.8 ± 21.3	0.006
Ablation lesions	length (mm)	10/10	5.0 ± 1.3	2.8 ± 0.2	0.000
	width (mm)	10/10	4.6 ± 0.7	4.2 ± 0.5	0.142
	height (mm)	10/10	4.2 ± 0.6	2.3 ± 0.4	0.000

##### Bipolar ablation in vitro

As shown in Table 3, bipolar ablation uniformly resulted in continuous ablation lesions with a length, width and depth of 2.8 ± 0.2, 4.2 ± 0.5, and 2.3 ± 0.4 mm, respectively (Table 3, Fig. 3).

### In vitro results of bipolar ablation and unipolar ablation

The in vitro ablation results were summarized in Table 3. Of the 4 study subjects, two were unipolar ablation, the others were bipolar ablation. There were significant differences for ablation characteristics and ablation lesions between bipolar ablation and unipolar ablation. Unipolar ablation could create greater lesion length and height (all *P* < 0.01). So, unipolar ablation is more effective than bipolar ablation using this CIDMA catheter.

## Discussion

This is the first study to report the main requirement and design process of CIDMA catheter, the feasibility and safety of the assembled catheter in vitro studies.

### The challenge of designing

The CIDMA catheter was designed to mimic the Lasso catheters, used for multi-electrode ablation and mapping in the pulmonary veins. One of the most challenging of designing was to ensure that the adjustable circle could be irrigated and driven independently. Therefore, ten 0.1 mm perfusion apertures, and two grooves were laser cut to produce the circle-irrigated structures, which were assembled and actuated by pull-wires.

Next was that the adjustable circle could be irrigated and ablated simultaneously. So, each of the electrodes possesses a thermocouple and 10 perfusion apertures arranged in two rows for irrigation.

The difference in stiffness between each segment should be an important issue of consideration. A nitinol rod was placed in an additional groove and could provide additional stiffness [14].

Then, a CIDMA catheter was assembled. In the plastic model and animal model, the pushability, torquability, and kink resistance were shown to be satisfactory. Depending on the anatomy, addressing the common pulmonary vein in the left side by the catheter was hampered frequently. By minimizing the diameter, bending and rotating the catheter tip, the catheter could enter pulmonary vein smoothly. As described previously [14], the handle could also present an effective holding and compensation mechanism, could ensure practical operation of the catheter, and allowed the actuation of the circled structure to mimic the target shapes. However, the stiffness of both the shaft and the tip shape of the assembled catheter were higher than regular catheters. Therefore, further studies are needed to define the suitable shaft stiffness by trading-off the required stiffness, enabling to steer the tip without bending the shaft of the catheter.

### Feasibility test in vitro

In vitro, our findings showed that ablation could produce obvious ablation lesions, and unipolar ablation was more effective than bipolar. Uniformity in the shape of ablation lesions was seen around the electrodes. Ablation lesion depth on tissue with gross pathology was corresponding to tissue characterization map on bisection. Tissue characterization map reliably characterized ablated tissue up to depths of 3 mm [19].

Yamane et al. demonstrated the effectiveness of large-size Lasso catheters [20]. The diameter of the Lasso catheter was 25–30 mm. In contrast, we used CIDMA catheter with a larger loop (20–35 mm in diameter) and could performed the ‘en bloc’ ablation of the large PVs. In large vessels, when enlarging the diameter, the catheter would be easy to trap into the antral region, and the bad touch between catheter and issue would be presented. In small PVs, in view of the adverse effects of the overlapping electrodes [9], precaution must be taken not to deliver RF energy when shrinking the diameter of loop.

Our test also showed that the circled structure was visible under real-time 3D anatomic mapping, and had a high 3D inducting accuracy (0.6~ 1.5 mm). Navigation was possible in a cardiovascular plastic model and an animal with an Ensite Velocity system.

Taken together, comparing with nMARQ catheter, the CIDMA catheter has more improved designs, and has many advantages theoretically, such as, of 10 electrodes, every single electrode has uniform perfusion, and uniform power distribution, and ablation lesions of 10 electrodes along the ablation line are size consistent. But further in vivo studies in animal AF models are needed to test these advantages.

## Conclusions

In vitro, our preliminary data suggest that the CIDMA catheter is feasible to produce optimal ablation lesions, especially through the unipolar ablation mode. Future in vivo animal and clinical studies are warranted to test the efficacy of this catheter in real world scenario.

## Abbreviations

3D: Three-dimensional
AF: Atrial fibrillation
CIDMA: Circular-irrigated, deca-channel mapping and ablation catheter, PEEK Polyether ether ketone
PVAC: Pulmonary vein ablation catheter
PVI: Pulmonary vein isolation
PVs: Pulmonary veins
RF: Radiofrequency
